# Enhancing Antibacterial Properties of Titanium Implants through Covalent Conjugation of Self-Assembling Fmoc-Phe-Phe Dipeptide on Titania Nanotubes

**DOI:** 10.1021/acsami.4c13885

**Published:** 2024-10-31

**Authors:** Ramesh Singh, Ketul C. Popat

**Affiliations:** † Department of Bioengineering, College of Engineering and Computing, George Mason University, Fairfax, Virginia 22030, United States; ‡ Department of Mechanical Engineering, Colorado State University, Fort Collins, Colorado 80523, United States

**Keywords:** Titania nanotubes, Fmoc-FF dipeptide, Antibacterial surface, Biomedical implants, Bacterial adhesion, Biofilm formation

## Abstract

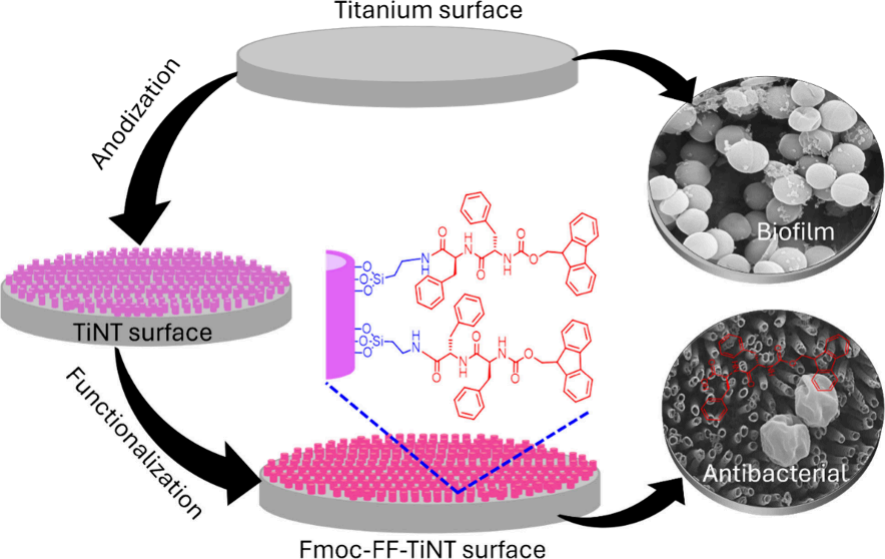

Bacterial infections and biofilm formation are significant challenges for medical implants. While titanium nanotube engineering improves biocompatibility, it cannot prevent bacterial adhesion and biofilm formation. Optimizing the biomaterial’s surface chemistry is vital for its desired functioning in the biological environment. This study demonstrates the covalent conjugating of the self-assembling dipeptide N-fluorenylmethyloxycarbonyl-diphenylalanine (Fmoc-FF) onto titanium nanotube surfaces (TiNTs) without altering the topography. Fmoc-FF peptides, in conjugation with TiNTs, can inhibit biofilm formation, eradicate pre-existing biofilms, and kill bacteria. This functionalization imparts antibacterial properties to the surface while retaining beneficial nanotube topography, synergistically enhancing bioactivity. Surface characterization by XPS, FT-IR, EDS, and SEM confirmed the successful functionalization. Bacterial adhesion experiments showed a significantly improved antibacterial activity of the functionalized TiNT surfaces. This study opens future possibilities for associating biomedical applications such as cell–cell interactions, tissue engineering, and controlled drug delivery of multifunctional self-assembling short peptides with implant materials through surface functionalization.

## Introduction

Titanium is a versatile material widely used for biomedical implants due to its corrosion-protective titanium oxide (titania) layer, which improves biocompatibility.^1,2^ Engineering porous titania nanotubes on the titanium surface provide excellent biocompatibility and allow better integration with the surrounding bone tissue (osseointegration).^3−6^ The nanotubular architecture enables drug delivery capabilities for cardiovascular stents and other implants.^7−9^ These properties make titanium an excellent choice for medical implants, ensuring their long-term stability, integrity, and performance within the body.^10^ In orthopedics, titanium is extensively used in joint replacements for the hips, knees, shoulders, and spine.^11,12^ In dentistry, titanium screws are surgically implanted into a patient’s jawbone to hold artificial teeth.^13,14^ Titanium is also employed in the housings of pacemakers to ensure the reliability of these cardiac devices.^15^ Despite the advancements, bacterial infections are a primary challenge associated with titanium-based biomedical implants.^16,17^ These infections are initiated by bacterial adhesion and, subsequently, the formation of polymicrobial biofilms on the implant surfaces.^11,16,17^ Biofilms are particularly problematic as they exhibit increased antibiotic resistance, making them difficult to eradicate.^18,19^ The biofilms’ complex microbial composition and three-dimensional structure contribute to antibiotic tolerance, leading to severe inflammatory processes and potential implant failure.^18−20^

When a biomaterial is implanted in the body, its surface comes into contact with the biological system, triggering a response that can influence the material’s desired function. The topographical modifications somewhat inhibit some bacteria’s growth based on their morphology instead of their chemical properties.^3,21,22^ Therefore, to enhance the antibacterial capabilities of TiNT surfaces, it is necessary to modify their surface chemistry and be equipped with antibacterial properties. Researchers are exploring various approaches for surface functionalization, including metal coatings such as silver, copper, and zinc, which are known for their antibacterial properties.^23,24^ Antibiotic and polymer coatings are being investigated as potential methods to enhance the antibacterial properties of titania nanoarrays.^23,24^ However, the use of these metal coatings leads to adverse side effects and toxicity.^25^ For example, silver coatings have been associated with the development of argyria, which causes skin discoloration.^25,26^ Furthermore, a common challenge with many of the antibacterial coating approaches for titanium implants is that the antibacterial substances are often only physically absorbed or have weak interactions with the surface.^25,27,28^ Consequently, the antibacterial properties of the existing coatings deteriorate quickly over time. Diminishing their ability to provide long-term protection against bacterial biofilm formation on the implant makes it challenging to prevent peri-implant infections consistently.^24,25,27,29^

Ultrashort peptide-based nanomaterials are promising biomaterials with important biomedical applications, such as antibacterial activity,^30,31^ drug delivery,^32,33^ and tissue regeneration.^34,35^ These peptides comprise 2–8 natural amino acid residues and have demonstrated remarkable antimicrobial properties, making them a compelling alternative to traditional antibiotics.^34,36,37^ Compared to conventional antibiotics, these peptide-based materials offer several advantages, including ease of synthesis, programmable assembly, reduced resistance risk, biocompatibility, and tunable activity.^34,36,38^ With their innate biocompatibility and distinctive self-assembling characteristics, short peptides are adaptable biomaterials for various biomedical uses, including antibacterial functions.^34,39^ The self-assembly of peptides containing the phenylalanine (Phe) motif, such as Di-l-phenylalanine (FF) and its Fmoc-protected Phe-Phe (Fmoc-FF), has been extensively studied and is a widely used approach for the formation of various nanobiomaterials for tissue engineering and drug delivery.^40,41^ The Fmoc and FF play a crucial role in self-assembly, as they can participate in π–π stacking interactions.^41−44^ This study was designed to improve the antibacterial activity of the titania nanotube surface by covalent conjugation of antibacterial dipeptides, Fmoc-protected Phe-Phe (Fmoc-FF). Dipeptide Fmoc-FF has demonstrated potent antimicrobial activities.^45−47^ Fmoc-protected amino acids and peptides, especially l-phenylalanine, have reported strong antibacterial and antibiofilm activity.^48−50^ Fmoc-Phenylalanine was reported as an antibiofilm formed in *S. aureus* and *P. aeruginosa*, clinically relevant bacteria in medical implants.^48^ The dipeptides Fmoc-FF and FF can induce membrane disruption and disrupt bacterial cell membranes, leading to cell death and inhibition of biofilm formation.^45−47^

## Results and Discussion

**Scheme 1:**
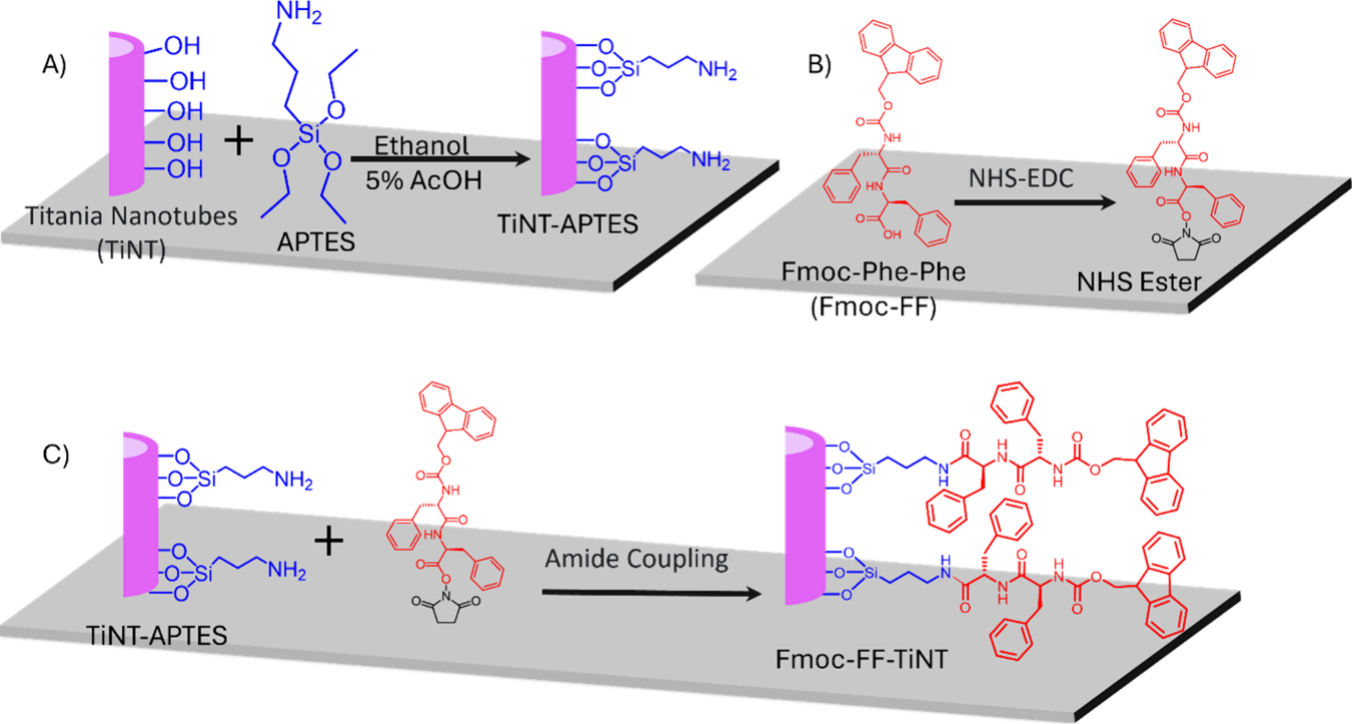
Reaction Scheme of Covalent Conjugation of Fmoc-FF on Titania Nanotube Surfaces. (A) Conjugation of APTES on the TiNT Surface Functionalized It, Free Amine Group, for Amide Coupling; (B) NHS-EDC Activation of the Carboxylic Group Followed; (C) Amide Coupling Results in Final Product Fmoc-FF Functionalized TiNT

The synthesis of titania nanotube surfaces (TiNT) on the titanium surfaces was achieved through the established laboratory procedure involving electrochemical anodization followed by annealing.^10,51,52^ The formation of TiNT surfaces was confirmed by visualizing them with scanning electron microscopy. Scheme 1 represents the covalent conjugation of Fmoc-FF-OH dipeptide, which was carried out in a two-step process: initially attaching (3-Aminopropyl)-triethoxysilane (APTES) having an amine group,^53,54^ followed by NHS-EDC amide coupling with the peptide.^55,56^ The conjugation of the peptides over titania nanotube surfaces has been confirmed with the help of surface characterization techniques, Fourier-transform infrared (FT-IR) spectroscopy, X-ray photoelectron spectroscopy (XPS), and energy-dispersive X-ray spectrometry (EDS).

**Figure 1:**
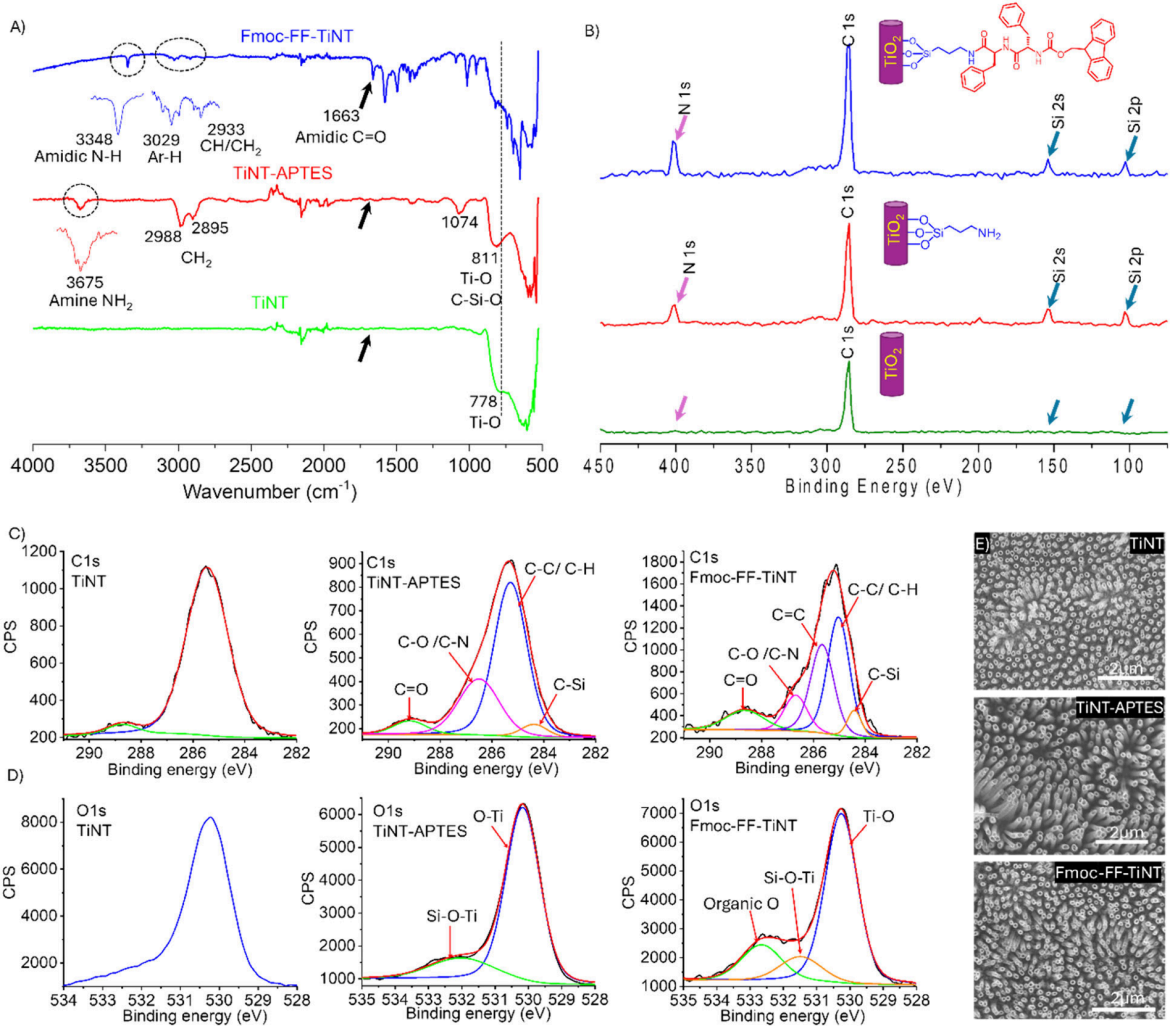
Characterization of functionalized titania nanotube surfaces. (A) FT-IR Spectra of the TiNT (green), TiNT-APTES (red), and Fmoc-FF-TiNT surfaces (blue). (B) Survey XPS spectra for different surfaces: titania nanotube arrays (TiNT, green), (3-Aminopropyl)-triethoxysilane (APTES) conjugated titania nanotube surfaces (TiNT-APTES, red), and Fmoc-Phe-Phe conjugated titania nanotube nanotubes (Fmoc-FF-TiNT, blue). (C and D) High-resolution XPS spectra for the C 1s and O 1s region, respectively, obtained from the different surfaces (TiNT, TiNT-APTES, and Fmoc-FF-TiNT) from left to right; and (E) representative SEM images of TiNT, TiNT-APTES, and Fmoc-FF-TiNT showing no morphological changes on functionalization of TiNT surfaces.

The FT-IR spectra were recorded for functionalized and nonfunctionalized TiNT surfaces to investigate the functional groups on the titania nanotube surfaces (Figure 1A) due to the conjugation of APTES and Fmoc-FF. A characteristic broadband for TiO_2_ from 895 to 520 cm^–1^ centered at 605 cm^–1^ was found for nonfunctionalized TiNT surfaces with a shoulder around 778 cm^–1^ (Figure 1A, green spectra).^57,58^ An amine group (−NH_2_) was evident upon functionalization with APTES, as indicated by an IR band at 3675 cm^–1^, and aliphatic −C–H stretching peaks were observed at 2988 and 2995 cm^–1^ (Figure 1A, red spectra).^56^ Additionally, the peak for Ti–O around 778 cm^–1^ shifted toward a higher wavenumber 811 cm^–1^ (a vertical line in Figure 1A indicating the shift), indicating the involvement of the Si–O bond with Ti–O and formation of the Si–O–Ti linkage on the surface.^57^ The observation of functional groups −NH_2_, CH_2_, and Si–O indicates the attachment of the silane linker onto the TiNT surfaces. After amide coupling was performed on the TiNT-APTES surface with Fmoc-FF dipeptide, the peak for amine disappears, and a peak at 3348 cm^–1^ arises for amidic -NH-, which differs in nature (singlet) from that for amine (multiplet), indicating the formation of an amide bond (Figure 1A, blue spectra). An aromatic C–H stretching peak due to phenyl rings at 3029 cm^–1^ and an aliphatic −C-H stretching band at 2933 cm^–1^ were also detected.^56^ A characteristic band in the amidic bond I region found for carbonyl (C=O) stretching (indicated with an arrow, 1663 cm^–1^) is absent in TiNT and TiNT-APTES. Along with these important bands for different functional groups, a notable change also appears in the TiO_2_ region.^59^ Overall, the FT-IR analysis indicates the presence of various functional groups on the TiNT surfaces, which means that Fmoc-FF has been conjugated.

Further, to confirm the binding of peptides to the titania surface, X-ray Photoelectron Spectroscopy (XPS) was utilized (Figure 1B and Figure S1).^60^ As expected, the characteristic peaks of Titanium and oxygen appeared for all three tested surfaces. Intense peaks for titanium at 459.9 eV (Ti 2p3) and 464.8 eV (Ti 2p1) and for Oxygen (O 1s) at 530.4 eV (Figure 1B).^57,60^ A peak for carbon (C 1s) at 284.8 eV also appeared in all three surfaces: in TiNT-APTES and Fmoc-FF-TiNT due to carbon present in different chemical states; however in the nonfunctionalized titania surface, it may be due to environmental carbon. A peak appears at 400.8 eV for nitrogen (N 1s) in the XPS spectra of TiNT-APTES and Fmoc-FF-TiNT, which was absent in the TiNT surfaces, supporting the functionalization of TiNT surfaces (Figure 1B, indicated by pink arrows). Additionally, peaks at 154.1 eV (Si 2s) and 102.7 eV (Si 2p) for silicon in the XPS of TiNT-APTES and Fmoc-FF-TiNT confirmed the functionalization (Figure 1B, indicated by green arrows).

For more detailed information about the chemical states of different elements observed in the XPS spectra, high-resolution XPS spectra were recorded for specific regions, including Ti 2p, O 1s, N 1s, C 1s, and Si 2p. The high-resolution XPS spectra for Ti 2p were consistent across all samples (Figure S2 A). Notably, changes were observed in the C 1s and O 1s regions, displaying composite peaks for functionalized TiNT surfaces (Figure 1 C and D). The deconvolution fit of the oxygen spectra revealed two components at 530.2 and 532.2 eV attributed to Ti–O and Si–O bonds in TiNT-APTES (Figure 1 D). In the case of Fmoc-FF -functionalized TiNT exhibited three oxygen components, two at 530.2 and 531.5 eV, contributed to Ti–O and Si–O bonds similar to TiNT-APTES, and the additional component at 532.66 eV for organic oxygen from peptides. Furthermore, deconvolution of the C 1s peak unveiled distinct peaks for the TiNT-APTES and Fmoc-FF-TiNT samples (Figure 1 C). For TiNT-APTES, peaks at 284.4, 285.2, 286.5, and 289.1 eV were assigned to C–Si, C–C, C–N, and C=O bonds, respectively. In comparison, Fmoc-FF-TiNT displayed peaks at 284.6, 285.0, 285.6, 286.7, and 288.6 eV corresponding to C–Si, C–C, C=C, and C–N/C–O bonds, respectively.^57,60^

Moreover, the N 1s and Si 2p peaks for TiNT-APTES were deconvoluted into two peaks each; N 1s exhibited peaks at 400.0 and 401.8 eV for amine and protonated amine, respectively (Figure S2 C), while Si 2p showed peaks at 101.6 and 102.7 eV for the O–Si–C and the O–Si–O bonds (Figure S2 B). In contrast, the peptide-conjugated TiNT displayed three components for N 1s at 398.3 400.2, and 401.4 eV, representing amide, amine, and protonated amine, respectively (Figure S2C). The Si 2p peak deconvolution mirrored that of TiNT-APTES with the identified O–Si–C and O–Si–O bonds in both cases (Figure S2 B). The peaks observed for Fmoc-FF conjugation TiNT surfaces in the XPS spectra are significant indicators of the surface chemical composition changes due to peptide attachment. Further, the emergence of a nitrogen peak at 400.8 eV (N 1s) and peaks at 154.1 eV (Si 2s) and 102.7 eV (Si 2p) for silicon after modification with APTES and Fmoc-FF conjugation signifies the presence of nitrogen and silicon elements on the titania surface. These peaks provide concrete evidence of peptide attachment on the titania surface, confirming the success of the modification process.

Further, to assess the durability of Fmoc-FF-TiNT surfaces, they were subjected to a water immersion test at 35 °C and subsequently examined using XPS. The XPS survey, conducted over a three week period, revealed that the atomic percentages of key elements, including silicon and nitrogen, remained largely consistent. These findings suggest that the peptide is firmly anchored to the titania nanotube surface, demonstrating a stable conjugation. This stability indicates that the Fmoc-FF-TiNT surfaces are suitable for long-term use under physiological conditions.

**Figure 2:**
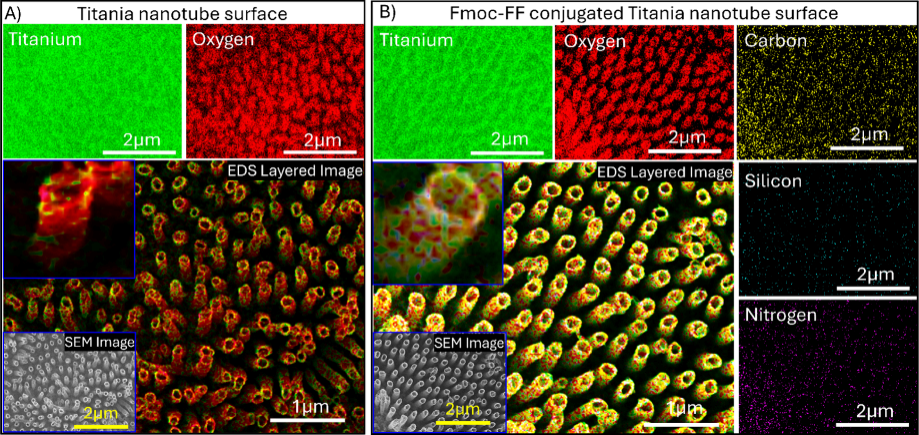
Energy-dispersive X-ray spectrometry (EDS) color map images that illustrate the elemental distribution on the surface of titania (TiNT) before and after functionalization. The image on the left (A) depicts the elemental composition of the bare titania surface with the color-coded elements being titanium (green) and oxygen (red). The image on the right (B) shows the titania surface after covalent conjugation of the dipeptide Fmoc-FF (TiNT-CES-TAN), where the color-coded elements are titanium (green), oxygen (red), carbon (yellow), silicon (cyan), and nitrogen (magenta). The layered image demonstrates the homogeneous distribution of the dipeptide on the titania surface, and the inset provides a close-up view of the functionalized TiNT surfaces.

After XPS and FT-IR confirmation of the successful covalent conjugation of Fmoc-FF on the surface of TiNT nanotube surfaces, SEM analysis was utilized to evaluate how the morphology of the TiNT surfaces is influenced. The SEM images indicate that the short peptide binds to the titania without causing any significant changes to its overall topography (Figure 1E). Furthermore, energy-dispersive X-ray spectrometry (EDS) was employed for elemental distribution on the titania nanotubes surfaces. The carbon and nitrogen peaks were observed in the EDS spectrum of the peptide-functionalized sample, albeit challenging to distinguish due to their overlap with oxygen and titania peaks (Figure S3). However, a silicon peak in the EDS spectra of functionalized TiNT surfaces supports the peptide attachment (Figure S3). Furthermore, the EDS elemental color map for functionalized and nonfunctionalized TiNT surfaces graphically illustrates the elemental distribution. The EDS analysis of TiNT predominantly displayed titanium (green) and oxygen (red) elements in its layered image (Figure 2A). At the same time, the peptide-functionalized TiNT exhibited distributions of five components: titanium (green), oxygen (red), carbon (yellow), Silicon (cyan), and nitrogen (magenta). The layered image displayed a combination of these colors (elements) on the titania nanotubes’ outer and inner surfaces (Figure 2B). The EDS color mapping corresponded well with the results obtained from spectroscopy and strongly supported the functionalization of the titania nanotube surfaces.

The conjugation of short peptides was anticipated to improve the biocompatibility of TiNT surface implants without causing any cytotoxicity. Peptides are small biomolecules composed of amino acids, which are the metabolites of proteins. Peptides are known for their inherent biocompatibility, meaning that they are well-tolerated by the body and do not elicit harmful immune responses. To verify the cytotoxicity behavior of the Fmoc-FF conjugated titania surface, a Lactate Dehydrogenase (LDH) assay cytotoxicity test was performed. The LDH assay is a widely used technique to quantify cell death and membrane damage by measuring the release of the LDH enzyme from damaged cells. Adipose-derived stem cells (ADSC) were grown and seeded directly onto different surfaces. Cells seeded on polystyrene were used as a negative control and on polystyrene treated with Triton as a positive control for complete cell lysis. The bar graph of absorbance (Figure S4) showed that the LDH level for all tested surfaces, including the Fmoc-FF conjugated TiNT surfaces, was statistically similar to the negative control. This was significantly lower than that of the positive control, indicating no cytotoxicity of the Fmoc-FF-conjugated TiNT surfaces.

The covalent conjugation with an antibacterial dipeptide alters the surface chemistry of the titania implant surfaces. The topographical effects of TiNT surfaces and Fmoc-FF functionality can boost the antibacterial effect of titania implants. An antibacterial assay was performed to assess the antibacterial efficacy of these functionalized titania nanotube surfaces. Among Gram-positive bacteria, *S. aureus* plays a major role in bacterial adhesion and subsequent biofilm formation on medical implant surfaces.^61−63^*P. aeruginosa*, a Gram-negative strain, is also known as a threat to biofilm formation in implantology.^64^ Therefore, these Gram-positive and Gram-negative bacteria strains were selected to investigate bacterial adhesion on the titania surface. A two-time point experiment at 6 and 24 h of bacterial adhesion was performed and imaged with fluorescence microscopy. After the culture time points were completed, the bacteria on the different surfaces were stained with two fluorescence dyes for imaging. SYTO 9 was used for live bacteria (green), and propidium iodide (PI) was used for dead bacteria (red) staining.

**Figure 3:**
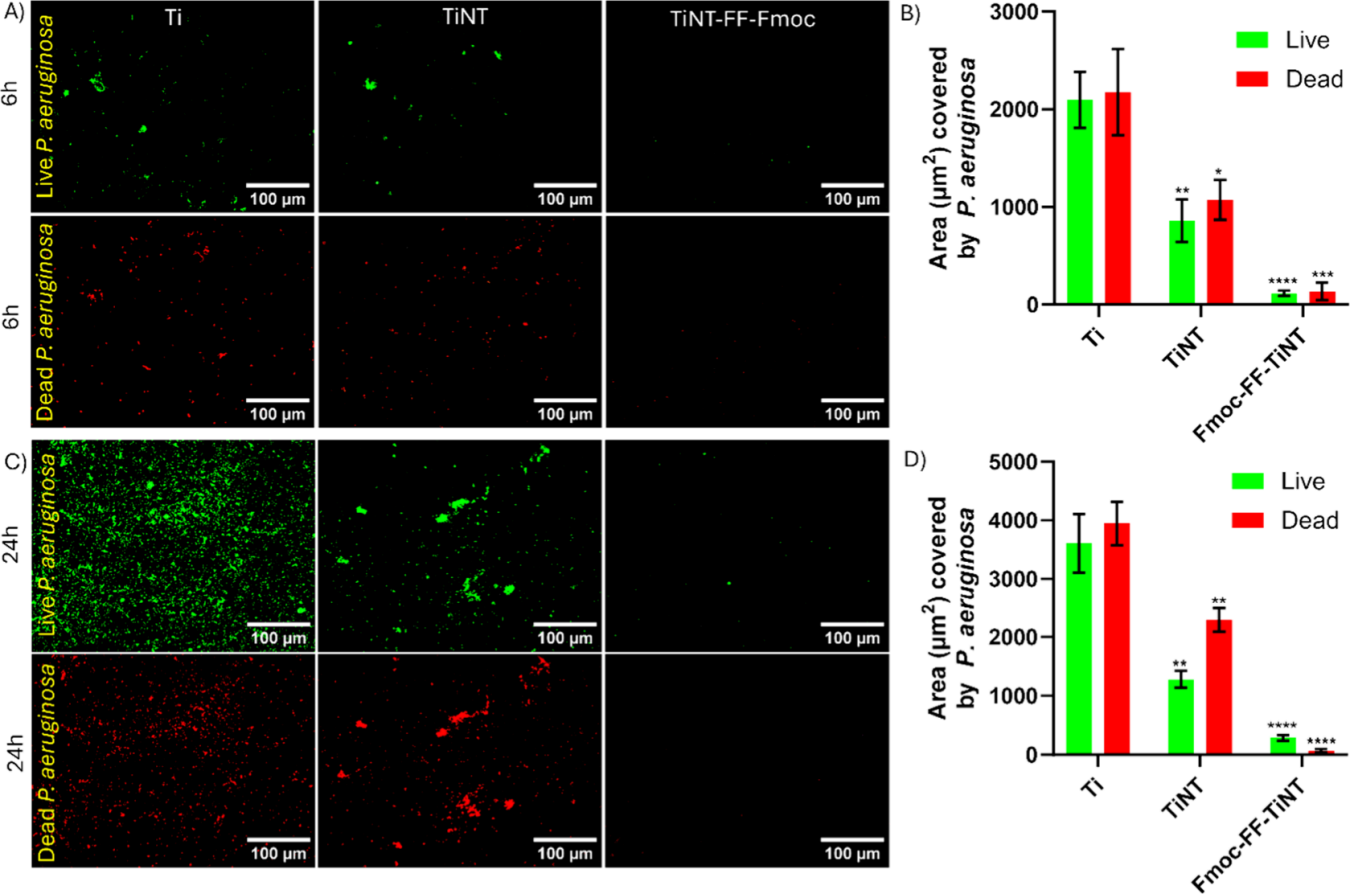
Representative fluorescence microscopy images of live (green) and dead (red) bacteria adhered on different surfaces: titanium (Ti), TiNT (TiNT), and Fmoc-FF-conjugated titania nanotube surfaces (Fmoc-FF-TiNT). (A) The adhesion of *P. aeruginosa* bacteria after 6 h of culture and (B) its corresponding quantification of the area covered by the adhesion of *P. aeruginosa* bacteria. (C) The adhesion of *P. aeruginosa* bacteria after 24 h of culture and (D) its corresponding quantification of the area covered by the adhesion of *P. aeruginosa* bacteria. Error bars indicate the mean with SD and the statistical significance (*p*-value) obtained compares live to live and dead to dead using a two-tailed unpaired *t* test, with the following levels of significance: ****p < 0.0001, ***p < 0.001, **p < 0.01, and *p < 0.05.

The fluorescence microscopy imaging revealed that the Fmoc-FF-functionalization of the titania surface exhibited a notable reduction in *P. aeruginosa* bacterial adhesion compared to that of the titanium and titania nanotube surfaces (Figure 3). The fluorescence images showed that live (green) and dead (red) bacteria equally adhered on the titanium surface, while in the case of TiNT surfaces, the concentration of dead bacteria was increased. The observation of dead bacteria on TiNT surfaces indicates the topographical effect and contact killing of *P. aeruginosa*. At the same time, the Fmoc-FF-conjugated titania surface showed a significant reduction in bacterial adhesion for both live and dead mice (Figure 3A and C). Quantifying fluorescence images with respect to the area covered by bacteria at 6 and 24h further supports the inhibition of *P. aeruginosa* on the Fmoc-FF functionalized titania surface (Figure 3B and D).

**Figure 4:**
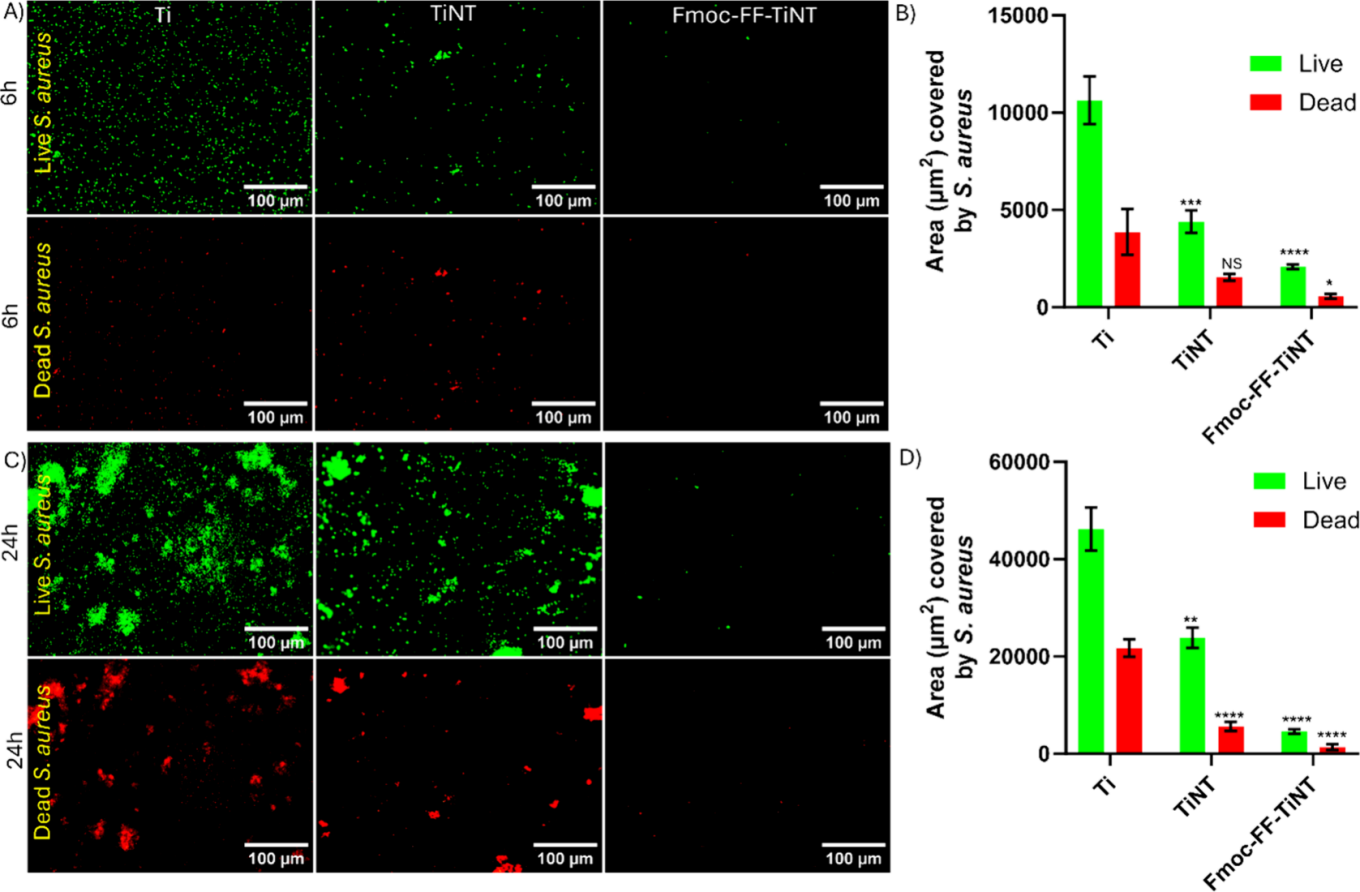
Representative fluorescence microscopy images of live (green) and dead (red) bacteria adhered on different surfaces: titanium (Ti), titania nanotube (TiNT), and Fmoc-FF-conjugated titania nanotube surfaces (TiNT-Phe-FF-Fmoc). (A) The adhesion of *S. aureus* bacteria after 6 h of culture and (B) its corresponding quantification of area covered by the adhesion of *S. aureus* bacteria. (C) The adhesion of *S. aureus* bacteria after 24 h of bacterial culture and (D) its corresponding quantification of area covered by the adhesion of *S. aureus* bacteria. Error bars indicate the mean with SD and the statistical significance (*p*-value) obtained compares live to live and dead to dead using a two-tailed unpaired *t* test, with the following levels of significance: ****p < 0.0001, ***p < 0.001, **p < 0.01, and *p < 0.05.

*S. aureus* is a major contributor to bacterial adhesion and biofilm formation. Fluorescence images also revealed a high growth of *S. aureus* bacteria on titanium and TiNT surfaces compared to *P. aeruginosa* bacteria. The bacterial growth was higher on the titanium surface than on TiNT surfaces for both the 6 and 24 h cultures (Figure 4). However, on the Fmoc-FF conjugated titania surface, bacterial growth was minimal. Quantification of the surface area covered by live and dead *S. aureus* bacteria colonies confirmed these trends for bacterial accumulation. The bar graphs clearly showed significant inhibition of bacterial colonization on the Fmoc-FF conjugated titania surface at the 6-h (Figure 4B) and *S. aureus* 24 h (Figure 4D) time points compared to the bare titania and titanium surfaces. The bacterial adhesion was quantified by measuring the fluorescent area in the image using ImageJ software. The results showed that the Fmoc-FF-TiNT surface had more than a 90% reduction in live and dead bacterial adhesion for both *S. aureus* and *P. aeruginosa* compared to the titanium surface.

**Figure 5:**
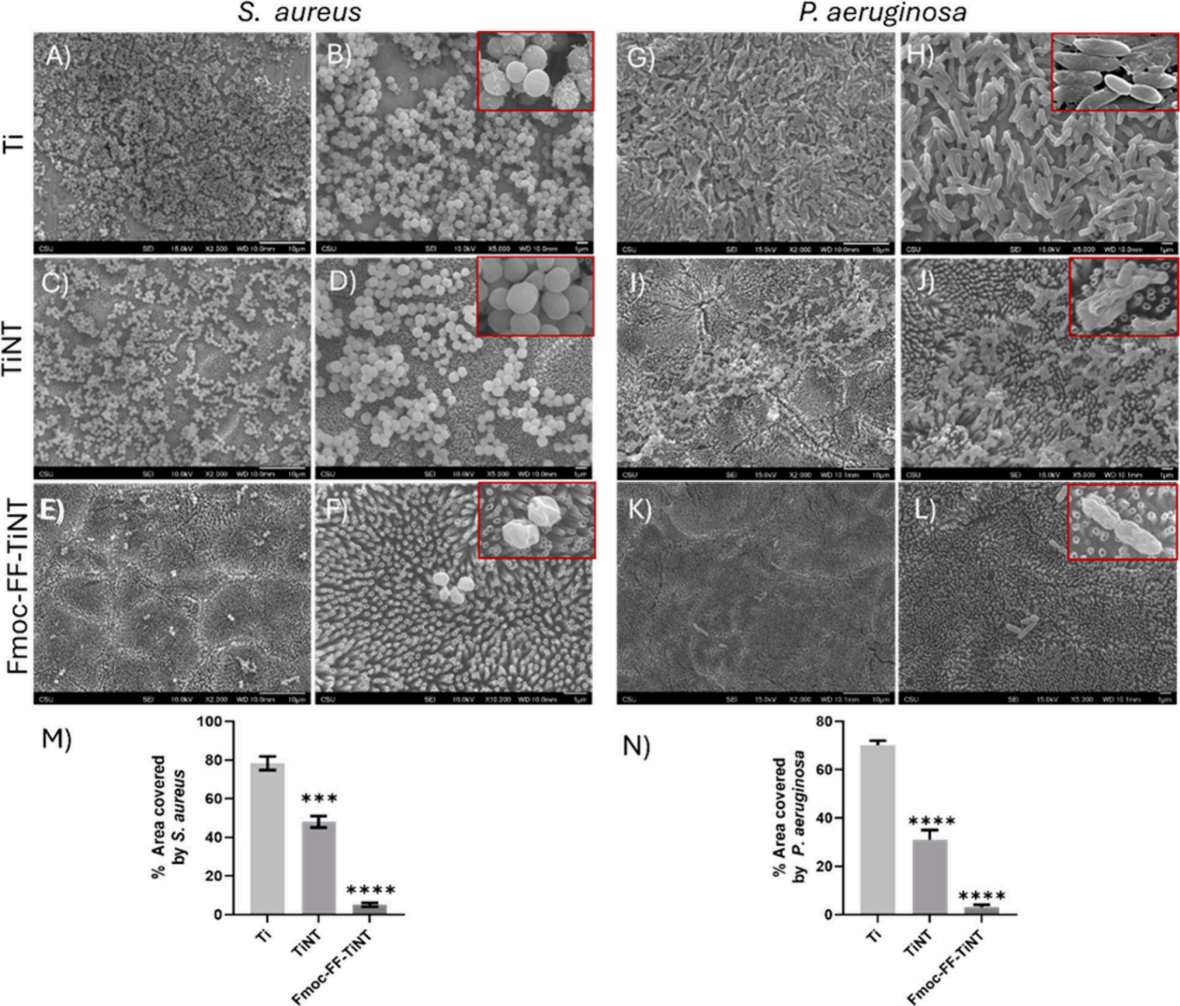
Representative Scanning Electron Microscope (SEM) images of bacterial adherence on functionalized and nonfunctionalized titania nanotube surfaces after 24 h of bacterial culture. *Left panel*: Depicts the aggregation of *S. aureus* on titanium (Ti) surfaces (A and B). The inset image shows the deposition of ECM-like material, indicating biofilm formation. (C and D) Titania nanotube (TiNT) surfaces and (E and F) Fmoc-FF-TiNT surfaces. The inset image shows the deformation of bacterial cell morphology, indicating the bactericidal property of Fmoc-FF-TiNT. *Right panel*: Depicts the aggregation of *P. aeruginosa* on titanium (Ti) surfaces (G and H). The inset image shows the aggregation of bacteria in the extracellular material, indicating biofilm formation. (I and J) Titania nanotube (TiNT) surfaces and (K and L) Fmoc-FF-TiNT surfaces. The inset image shows the deformation of *P. aeruginosa* cells, indicating the bactericidal effect of Fmoc-FF-TiNT. Quantifying the area covered by *S. aureus* (M) and *P. aeruginosa* (N), corresponding to the SEM images. Error bars indicate the mean with SD and the statistical significance (*p*-value) obtained using a two-tailed unpaired *t* test, with the following significance levels: ****p < 0.0001 and ***p < 0.001.

SEM imaging visualized the morphological nature of bacterial aggregation on the tested surfaces. For 6 h of bacterial culture, *P. aeruginosa* growth over the titanium surface is greater than other tested surfaces (Figure S5G-H). TiNT surfaces displayed a significantly lesser number of bacteria, as observed in fluorescence microscopy. The scanned area of Fmoc-FF functionalized TiNT surfaces has very few isolated bacteria, reflecting the inhibitory potential of peptide functionalization (Figure S5K-L). *P. aeruginosa* is generally known for colonizing medical devices and human tissues and for forming an antibacterial-resistant biofilm. SEM imaging of *P. aeruginosa* after 24 h of bacterial cultures revealed its aggregation and multilayered colonization embedded in an extracellular matrix over the titanium surface, indicating the starting of a biofilm formation (Figure 5G-H). With a little reduction, biofilm retention was observed on the nanoengineered TiNT surface (Figure 5I-J). The conjugation of the self-assembling, phenyl alanine-based antibacterial dipeptide Fmoc-FF on the titania surface remarkably inhibited bacterial aggregation, preventing biofilm formation (Figure 5K-L). The SEM images of *P. aeruginosa* on functionalized TiNT surfaces showed a few isolated bacteria.

The biofilm protection of bacteria from the host defense system and antibiotics results in antibiotic resistance. Around 80% of bacterial infections associated with medical implants are due to *S. aureus* adherence.^61−63^ The SEM investigation of *S. aureus* cultures over different tested surfaces showed the highest bacterial adherence on titanium surfaces, followed by that on TiNT surfaces. SEM imaging at the 6 h bacterial culture revealed a similar trend for *S. aureus* as observed with *P. aeruginosa*. However, the aggregation of *S. aureus* was greater than that of *P. aeruginosa* on the titanium surface. Interestingly, the SEM imaging of Fmoc-FF functionalized TiNT surfaces unveiled a negligible bacterial count on the surface (Figure S5A-F). After a 24 h culture of *S. aureus*, SEM imaging revealed that bacteria almost entirely covered the titanium surface and started growing into three dimensions (Figure 5A-B). The high-resolution SEM revealed some material deposition over the bacterial surface, indicating the release of an extracellular matrix, which can eventually result in biofilm formation (Figure 5B). A nearly identical imaging was observed for TiNT surfaces with a slight reduction in aggregation. However, no bacterial aggregation or biofilm was found on the Fmoc-FF functionalized TiNT surfaces (Figure 5E-F). Furthermore, the high-resolution SEM images revealed that the bacteria on Fmoc-FF-TiNT had compromised their structures, indicating bacterial killing by functionalized TiNT (Figure 5F and L).

**Figure 6:**
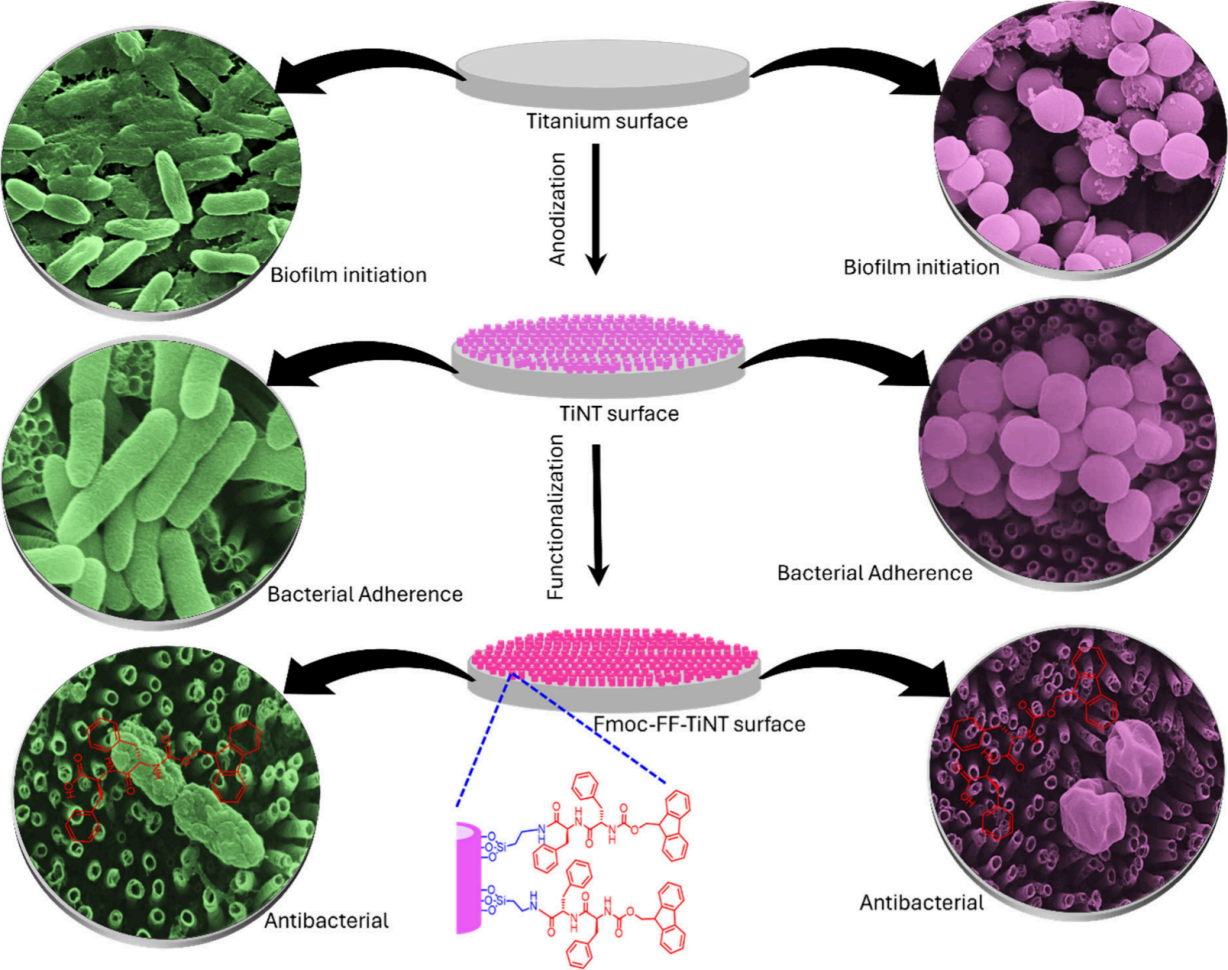
Schematic illustration of titanium surface modification and dipeptide grafting. This figure depicts the synergistic approach of modifying the titanium surface to create a porous nanotubular topography followed by the grafting of an antibacterial dipeptide. The combined effect of the nanostructured surface and the antimicrobial dipeptide functionalization aims to inhibit bacterial adherence and colonization on the titanium implant material.

Phenylalanine-based peptides with N-terminal Fmoc (9-fluorenylmethyloxycarbonyl) protection employ a multifaceted mechanism of action to inhibit bacterial growth.^45,46,48,49^ They disrupt the integrity of the bacterial cell membrane, leading to cell death. Fmoc-protected diphenylalanine (Fmoc-FF) peptides have been shown to induce oxidative and osmotic stress within bacterial cells, enhancing their antibacterial efficacy. Even at low concentrations, Fmoc-F residues can inhibit bacterial growth by reducing glutathione levels, which is an essential antioxidant in bacterial cells. These Fmoc-protected phenylalanine-based peptides and amino acids reduce extracellular matrix (ECM) components, such as proteins, carbohydrates, and DNA, inhibiting biofilm formation and eradicating pre-existing biofilms on various surfaces.^48,49,65,66^ On the other hand, the titania nanotube arrays have been shown to promote enhanced cell adhesion, proliferation, and differentiation of osteoblasts and bone marrow stromal cells, leading to improved osseointegration between the implant and the surrounding bone tissue.^7,8,67^ The titania nanotubes also demonstrate a certain degree of morphology-based contact killing of bacteria.^3,11,22^ Therefore, combining these peptides on a titanium surface with the retention of biomedical benefits of titania nanotubular modification^7,9,67^ can equip medical devices with multifunctional antibacterial ability.^8,9,22^ A schematic representation of the work summary here, represented in Figure 6, displays a combined effect of topography and peptides biomolecules.

Furthermore, short peptides have great potential in various applications in the biomedical field, including drug delivery, tissue engineering, and regenerative medicine.^4,34−37^ This study paves the way for developing next-generation multifunctional implant surfaces with improved antibacterial and biocompatibility characteristics. This study can be extended to conjugate short peptide-based biomaterials onto various medical implants and devices such as orthopedic, dental, and cardiovascular implants, wound dressings, and tissue engineering scaffolds. This approach can significantly reduce the risk of implant-associated infectious diseases in the peripheral, improve medical devices’ long-term performance and success, and ultimately enhance patient outcomes.

## Conclusions

This research study has demonstrated the successful covalent conjugation of the self-assembling dipeptide Fmoc-FF onto TiNT surfaces to enhance the antibacterial properties of biomedical implants. XPS and FT-IR confirmed the successful conjugation with new peaks corresponding to nitrogen, silicon, and various functional groups on the Fmoc-FF-TiNT surface. The antibacterial evaluation with Gram-positive (*S. aureus*) and Gram-negative (*P. aeruginosa*) bacteria revealed that the Fmoc-FF-TINT surfaces significantly inhibited bacterial adhesion and biofilm formation. This enhanced antibacterial efficacy was attributed to the synergistic effects of the topographical features of the nanotubes and the inherent antibacterial properties of the self-assembling Fmoc-FF dipeptide. The findings of this study highlight the potential of the Fmoc-FF-conjugated TiNT surfaces for developing advanced antibacterial coatings for a wide range of biomedical implants such as orthopedic and dental implants. Fmoc-FF peptides in conjugation with TiNT can inhibit biofilm formation, eradicate pre-existing biofilms, and kill bacteria through multiple mechanisms involving membrane disruption, oxidative stress, and interference with cellular processes. This approach can help prevent implant-associated infections and improve these medical devices’ long-term performance and success by inhibiting bacterial adhesion and biofilm formation.

## Materials and Methods

### Materials

The chemicals used in this research were obtained from reliable suppliers. Diethylene glycol (DEG) was purchased from Thermo Fisher Scientific Chemicals, Inc., while hydrofluoric acid (HF, 48%) was obtained from KMG Electronic Chemical. Additionally, the following compounds were acquired from Sigma-Aldrich: (3-Aminopropyl) triethoxysilane (APTES), 1-ethyl-3-(3-dimethylaminopropyl) carbodiimide hydrochloride (EDC), and *N*-hydroxysuccinimide (NHS). N-Fluorenylmethyloxycarbonyl-diphenylalanine (Fmoc-Phe-Phe) was purchased from Synthonix, Inc.

### Synthesis of Titania Nanotube Arrays

A commercially available medical-grade pure titanium sheet (0.5 mm thick) was used as the starting material to fabricate titania nanotube arrays (TiNT). The synthesis of the TiNT on the titanium surfaces was achieved through an established laboratory procedure involving electrochemical anodization followed by annealing.^6,51,68,69^ The detailed procedure includes:

#### 1 Surface Preparation and Cleaning before Titania Nanotube Engineering

Before the titania nanotube engineering process, the titanium foil was first polished using silicon carbide sheets and then cut into small pieces measuring 2 cm × 2 cm. These small pieces were then cleaned by sonication in acetone for 10 min, followed by a soap solution. The samples were further sonicated for 10 min in isopropyl alcohol and 10 min in deionized water (DI), then dried inside a fume hood.

#### 2 Anodization

The cleaned Ti surfaces were then anodized in an electrolyte solution of 2% hydrofluoric acid, 3% DI water, and 95% diethylene glycol (DEG) at 55 V for 22 h. A platinum foil was used as the cathode.

#### 3 Annealing

After anodization, the electrolyte solution was washed from the titanium surface with DI water and isopropyl alcohol and then dried. The dried anodized sheets were then annealed in an oven at 530 °C in an ambient oxygen environment for 3 h with a 15 °C/min temperature increment.

The formation of titania nanotube arrays was confirmed by SEM imaging. These synthesized TiNT surfaces are stored at room temperature and used for further modification.

### Functionalizing Titania Nanotube Surfaces with Fmoc-FF

A 2 × 2 cm^2^ titania nanotube sheet was treated with a solution of APTES in ethanol containing 5% acetic acid and stirred on a shaker for 5 h.^53,54^ Following the reaction, the sheet was rinsed with ethanol and air-dried. The successful functionalization was assessed using IR spectroscopy and further confirmed through X-ray Photoelectron Spectroscopy (XPS). Subsequently, the dried silanized sheet was utilized for peptide coupling. In a beaker, equimolar amounts of NHS and EDC were dissolved in DMF, followed by adding one equivalent of Fmoc-protected diphenyl alanine, Fmoc-FF–OH, and stirred for 2 h to activate the ester. The APTES-functionalized titania sheet was then immersed in this solution and allowed to react overnight (12–16 h).^55,56^ The peptide-linked sheet was washed with DMF, rinsed with ethyl alcohol, and dried. Confirmation of successful peptide linking was achieved through FT-IR analysis and XPS.

### Material Characterization

#### X-ray Photoelectron Spectroscopy (XPS)

The X-ray photoelectron spectroscopy (XPS) analysis was conducted using a PHI Physical Electronics PE-5800 X-ray Photoelectron Spectrometer installed at Colorado State University, Fort Collins, Colorado, USA. The instrument was equipped with an Al Kα X-ray source, which was used for collecting both survey and high-resolution XPS spectra of different surfaces. Survey spectra were collected for all the surfaces, covering the energy range from 0 to 1100 eV. The peak-fit analysis was performed using the MultiPak software. Elemental analysis was also done for each surface using MultiPak (version 9.6.1.7). The high-resolution spectra of different elements were analyzed and deconvolved by using the CASA XPS software. This allowed for a detailed examination of the chemical composition and bonding states present on the surfaces. The final data reports are plotted in Origin Pro software.

#### Fourier-Transform Infrared Spectroscopy (FT-IR)

The infrared (IR) spectra of functionalized and nonfunctionalized titania nanotube arrays were recorded using a Thermo Nicolet iS50 Fourier-transform infrared (FTIR) spectrometer equipped with an attenuated total reflectance (ATR) accessory. The spectral range scanned was from 4000 to 500 cm^–1^.

#### Scanning Electron Microscopy (SEM)

A JEOL JSM-6500F field emission scanning electron microscope (FESEM) installed at Colorado State University, Fort Collins, Colorado, USA, was used for morphological characterization at 5 to 15 kV. Energy-dispersive spectroscopy (EDS) spectra were collected for different surfaces using an Oxford SDD EDS detector connected with this FE-SEM. EDS color map imaging was performed to visualize the elemental distribution on the titania nanotube surfaces, and the EDS data were analyzed using Oxford Aztec software.

#### Evaluating the Cytotoxicity of Different Surfaces

To assess the cytotoxic effects of various surfaces on human adipose-derived stem cells (ADSCs), we conducted a lactate dehydrogenase (LDH) cytotoxicity assay was conducted. The CyQUANT LDH Cytotoxicity Assay Kit from ThermoFisher Scientific, Waltham, MA, USA, was used. ADSCs were cultured in a medium of 90% MEM Alpha Modification (1 × , Cytiva, Marlborough, MA, USA), 9% fetal bovine serum (FBS), and 1% penicillin-streptomycin. The surfaces to be tested were placed in a 48-well plate, sterilized under UV light for 30 min, and then washed with PBS. ADSCs were seeded directly onto these surfaces at 40,000 cells/mL density and incubated at 5% CO_2_ for 24 h. Cells cultured on polystyrene (PS) were used as the negative control for cytotoxicity, while cells on polystyrene treated with 1.0% Triton X for 45 min served as the positive control. After 24 h of incubation, the culture media from each well was collected and added to an equal amount of LDH substrate reagent solution (Quantichrom Bioassay Systems, Hayward, CA, USA) in a 96-well plate. The mixture was incubated for 30 min, and the absorbance of the LDH solution in each well was measured at 490 and 680 nm wavelengths using a plate reader (FLUOstar Omega, BMG LABTECH, Cary, NC, USA).). Five replicates of each surface (Ti, TiNT, and TiNT-TAN) were used in the experiment. Results are presented as mean ± standard deviation, and a two-tailed unpaired *t* test was used to compute *p*-values.

### Bacterial Culture and Adhesion Evaluation

#### Bacterial Culture

The antibacterial properties of the Ti, TiNT, and Fmoc-FF-TiNT surfaces were assessed with two bacterial strains: one Gram-positive *S. aureus* and the Gram-negative *P. aeruginosa*. These bacteria were cultured in tryptic soy broth (TBS) at 37 °C for 24 h until a bacterial concentration of 0.52 unit absorbance at 562 nm or 10^9^ colony-forming units (CFU)/mL was achieved. Then, these bacterial solutions are further diluted to 106 CFU/mL and seeded over different surfaces to evaluate the bacterial adhesion and morphology. The bacterial cultures were incubated for two-time points, 6 h and 24 h, at 37 °C. After the incubation, these tested surfaces were washed with PBS to remove any nonadhered bacteria.

#### Fluorescence Microscopy

Fluorescence microscopy was used to evaluate the viability and adhesion of bacteria on the tested surfaces. Each tested surface was incubated in a staining solution containing a 1:1 ratio of propidium iodide (a stain for dead bacteria) and Syto 9 (a stain for live bacteria) in PBS for 15 min at room temperature. After the staining, the bacterial cells were incubated on the surfaces in a 3.7% formaldehyde solution for 15 min. After washing with PBS, the stained and fixed surfaces were imaged using a fluorescence microscope. The acquired images were analyzed by using ImageJ software to quantify the percentage of the surface area covered by live and dead bacteria. Three replicates were performed for each surface, and at least three images were taken per sample (9/sample) for quantification. Results are presented as mean ± standard deviation and a two-tailed unpaired *t* test used to compute *p*-values.

#### Bacteria Morphology and Biofilm Formation

These tested surfaces were incubated in a primary fixative solution for 45 min for SEM investigation of adhered bacteria and biofilm formation analysis. The fixative solution contained 3% glutaraldehyde, 0.1 M sucrose, and 0.1 M sodium cacodylate in deionized water. The surfaces were then incubated in a buffer solution containing the fixative components, except for the glutaraldehyde, for 10 min. The surfaces were then washed with a series of ethanol solutions (35%, 50%, 70%, and 100%) with a 10 min incubation in each solution for dehydration. After dehydration, the surfaces were kept dry inside a desiccator. Before imaging with SEM, a 10 nm gold coating was applied to the surfaces to improve the surface conductivity for imaging.

## Supporting Information Available

Survey and high-resolution XPS, SEM images of bacteria on different surfaces, EDS spectra of functionalized and nonfunctionalized TiNT surfaces, and LDH assay results (PDF)
