# Supplementary material for: Enhancing Antibacterial Properties of Titanium Implants through Covalent Conjugation of Self-Assembling Fmoc-Phe-Phe Dipeptide on Titania Nanotubes
Source: ACS Appl Mater Interfaces. 2024 Oct 31;16(45):61714–24. doi: 10.1021/acsami.4c13885 (PMC11565481; doi:10.1021/acsami.4c13885)
Supplement: Supplementary file 1 — am4c13885_si_001.pdf [file am4c13885_si_001.pdf]

Supporting Information

Enhancing Antibacterial Properties of Titanium Implants through Covalent Conjugation of Self-Assembling Fmoc-Phe-Phe Dipeptide on Titania Nanotubes

Ramesh Singh[a] and Ketul C. Popat[a, b, *]

[a] Department of Bioengineering, College of Engineering and Computing, George Mason University, Fairfax, Virginia 22030 USA.

[b] Department of Mechanical Engineering, Colorado State University, Fort Collins, Colorado 80523 USA.

* Authors of correspondence: ketul.popat@colostate.edu and kpopat@gmu.edu

Figures:

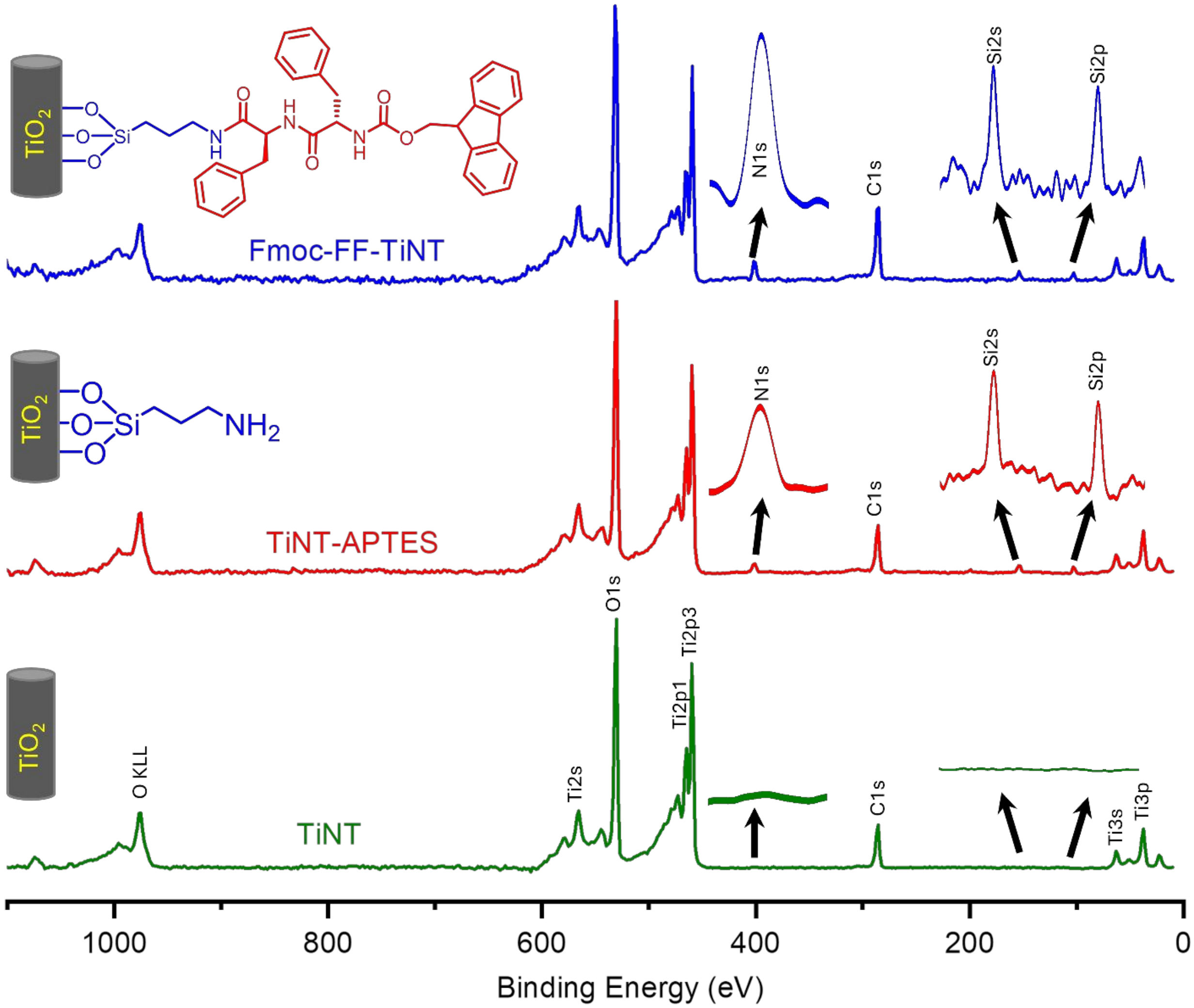

Figure S1: The Survey XPS spectra for different surfaces: titania nanotube arrays (TiNT, green), (3-Aminopropyl)-triethoxysilane (APTES) conjugated titania nanotube surfaces (TiNT-APTES, red), and Fmoc-Phe-Phe conjugated titania nanotube nanotubes (Fmoc-FF-TiNT, blue).

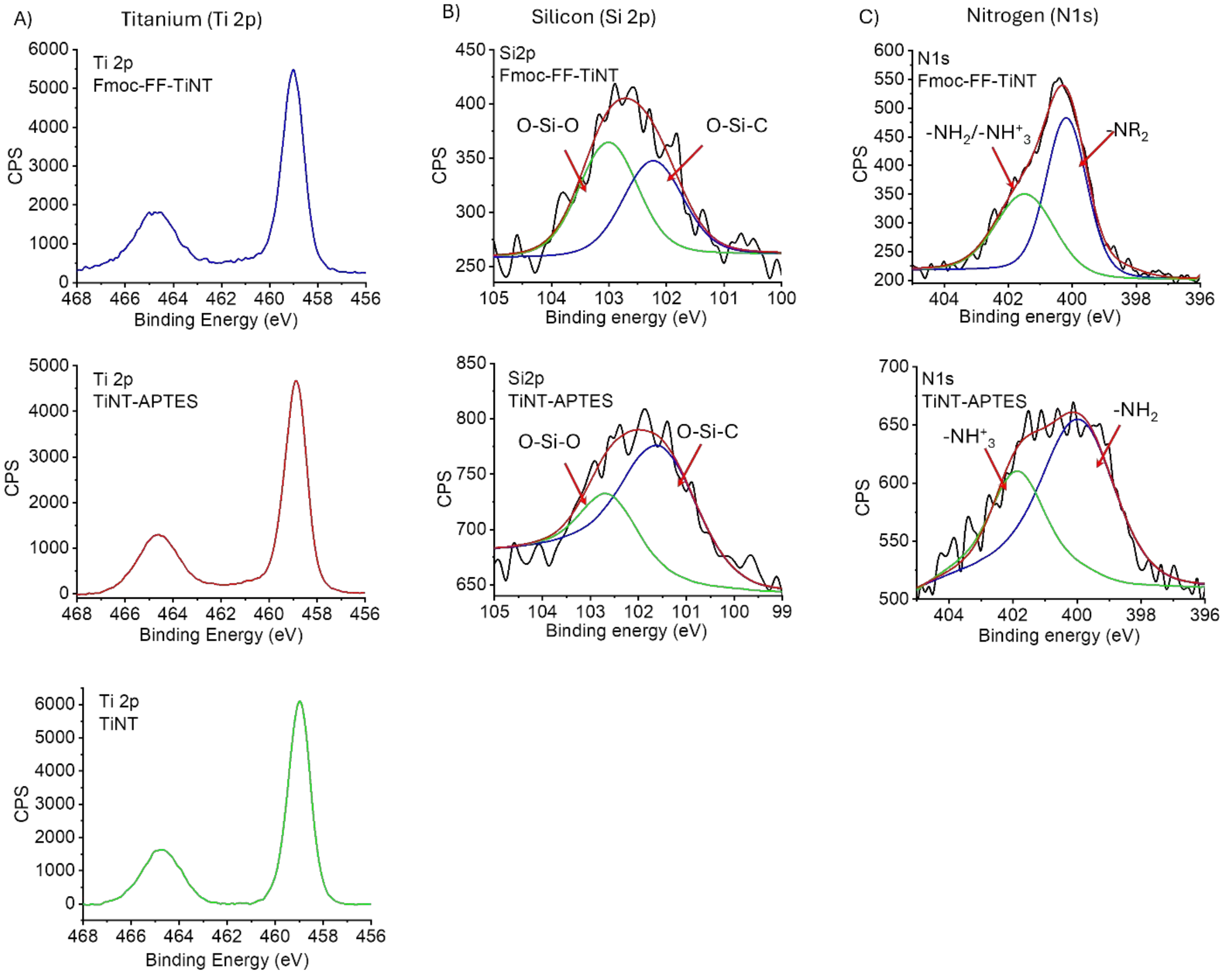

Figure S2: The High-resolution XPS spectra of A) Ti 2p, B) Si2p, and C) N 1s regions, obtained from the different surfaces surfaces (TiNT, TiNT-APTES, and Fmoc-FF-TiNT).

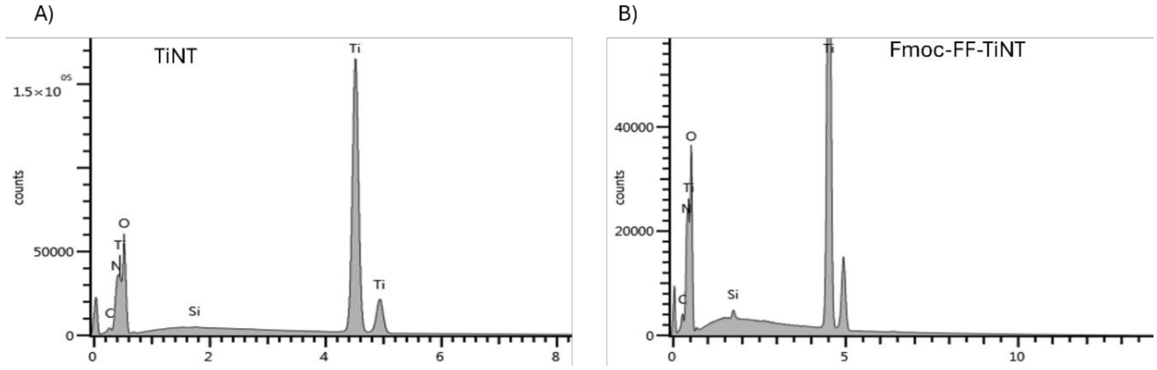

Figure S3: The energy dispersive spectroscopy (EDS) spectra of A) TiNT and B) Fmoc-FF-TiNT.

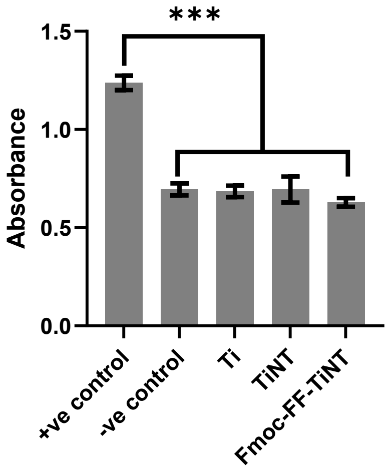

Figure S4: Bar graph for cytotoxicity results were assessed using LDH assay. Error bars indicate the mean with SD, and the statistical significance (p-value) obtained using a two-tailed unpaired t-test, significance ***p-value < 0.001.

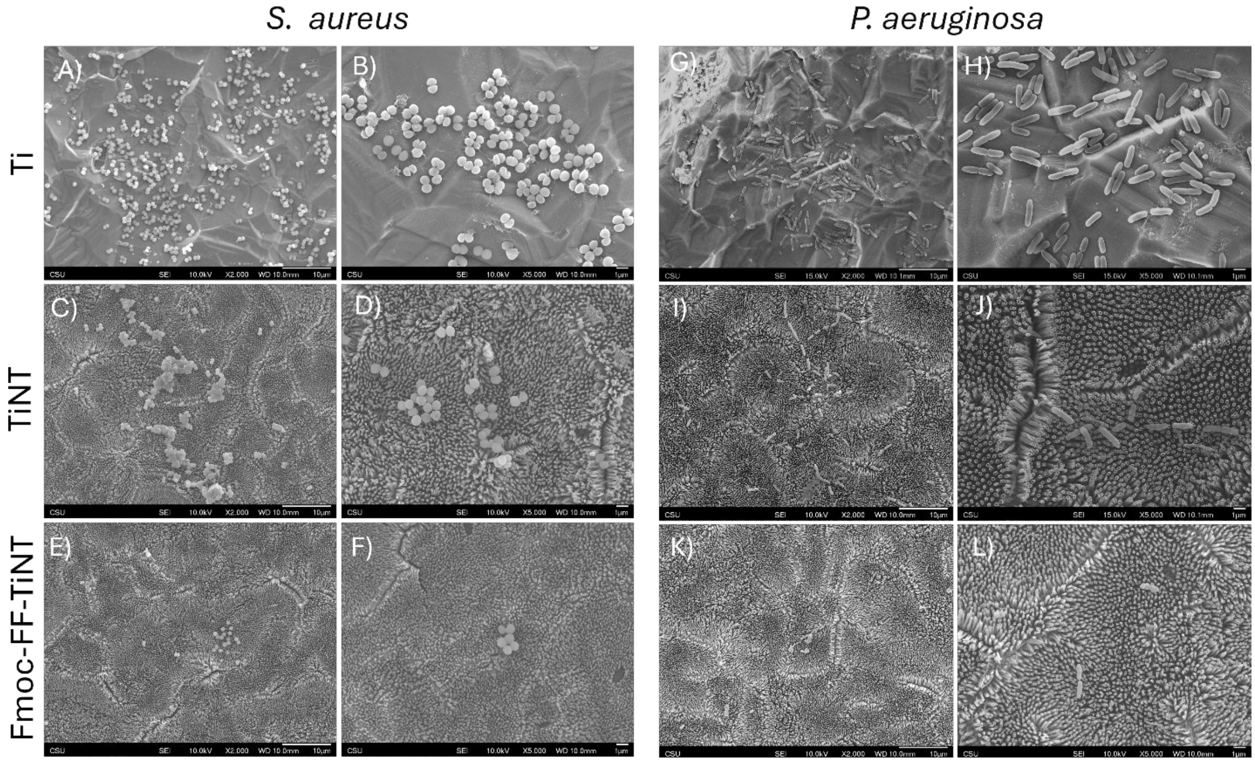

Figure S5: Representative Scanning Electron Microscope (SEM) images of bacterial adherence on functionalized and non-functionalized titania nanotube surfaces after 6 hours of bacterial culture. ***Left panel*:** Depicts the aggregation of *S. aureus* on Titanium (Ti) surfaces (A and B), Titania Nanotube (TiNT) surfaces (C and D), and Fmoc-FF-TiNT surfaces (E and F). ***Right panel*:** Depicts the aggregation of *P. aeruginosa* on Titanium (Ti) surfaces (G and H), Titania Nanotube (TiNT) surfaces (I and J), and Fmoc-FF-TiNT surfaces (K and L).

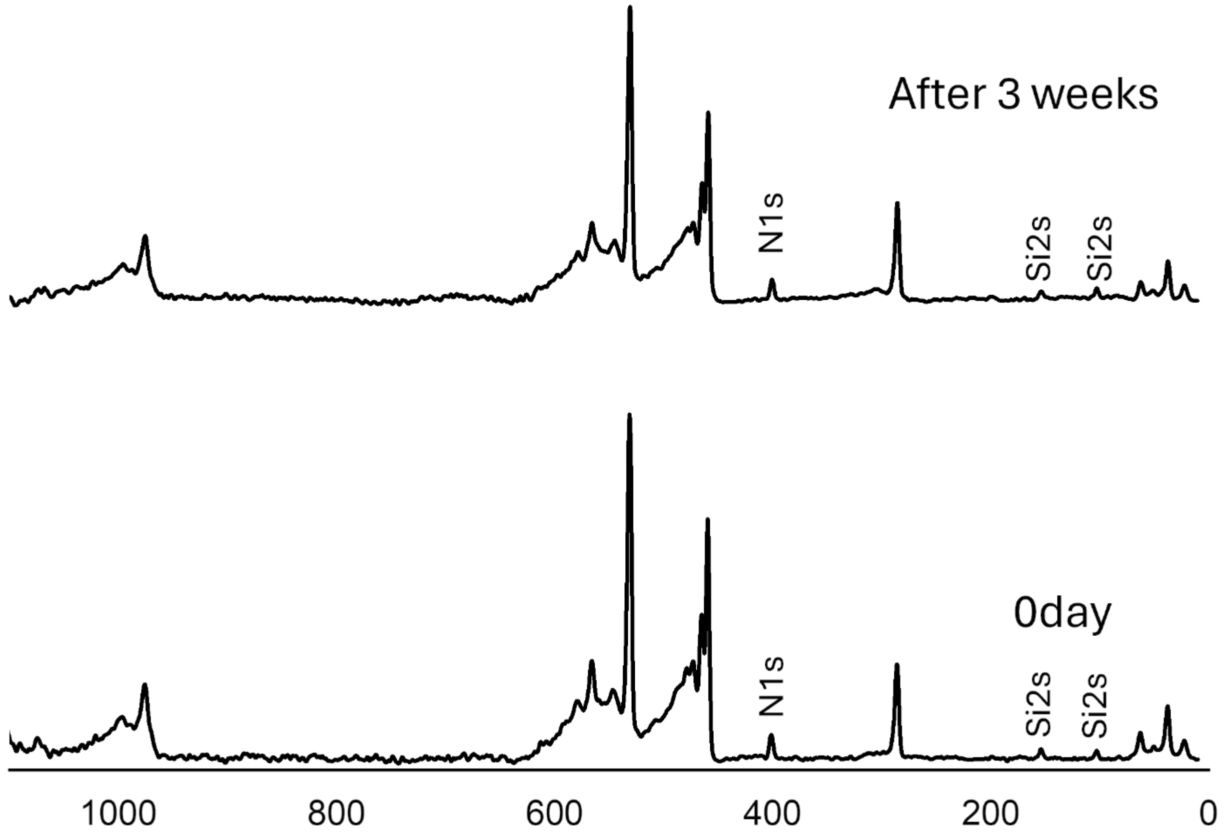

Figure S5: XPS survey spectra of Fmoc-TiNT surfaces after 0 and 3 weeks of water incubation. The presence of all peaks in both spectra demonstrates the durable adhesion of peptides to the titania nanotubes.
